# Natural Language Processing for Rapid Response to Emergent Diseases: Case Study of Calcium Channel Blockers and Hypertension in the COVID-19 Pandemic

**DOI:** 10.2196/20773

**Published:** 2020-08-14

**Authors:** Antoine Neuraz, Ivan Lerner, William Digan, Nicolas Paris, Rosy Tsopra, Alice Rogier, David Baudoin, Kevin Bretonnel Cohen, Anita Burgun, Nicolas Garcelon, Bastien Rance, AP-HP/Universities/INSERM COVID-19 Research Collaboration; AP-HP COVID CDR Initiative

**Affiliations:** 1 Department of Biomedical Informatics Necker-Enfant Malades Hospital Assistance Publique – Hôpitaux de Paris (AP-HP) Paris France; 2 Centre de Recherche des Cordeliers INSERM UMRS 1138 Team 22 Université de Paris Paris France; 3 LIMSI CNRS Université Paris Saclay Orsay France; 4 Department of Biomedical Informatics Georges Pompidou European Hospital Assistance Publique – Hôpitaux de Paris (AP-HP) Paris France; 5 DSI WIND Assistance Publique – Hôpitaux de Paris (AP-HP) Paris France; 6 School of Medicine University of Colorado Denver, CO United States; 7 Institut Imagine, INSERM U1163 Université Paris Descartes Université de Paris Paris France; 8 Please see acknowledgements for list of collaborators

**Keywords:** medication information, natural language processing, electronic health records, COVID-19, public health, response, emergent disease, informatics

## Abstract

**Background:**

A novel disease poses special challenges for informatics solutions. Biomedical informatics relies for the most part on structured data, which require a preexisting data or knowledge model; however, novel diseases do not have preexisting knowledge models. In an emergent epidemic, language processing can enable rapid conversion of unstructured text to a novel knowledge model. However, although this idea has often been suggested, no opportunity has arisen to actually test it in real time. The current coronavirus disease (COVID-19) pandemic presents such an opportunity.

**Objective:**

The aim of this study was to evaluate the added value of information from clinical text in response to emergent diseases using natural language processing (NLP).

**Methods:**

We explored the effects of long-term treatment by calcium channel blockers on the outcomes of COVID-19 infection in patients with high blood pressure during in-patient hospital stays using two sources of information: data available strictly from structured electronic health records (EHRs) and data available through structured EHRs and text mining.

**Results:**

In this multicenter study involving 39 hospitals, text mining increased the statistical power sufficiently to change a negative result for an adjusted hazard ratio to a positive one. Compared to the baseline structured data, the number of patients available for inclusion in the study increased by 2.95 times, the amount of available information on medications increased by 7.2 times, and the amount of additional phenotypic information increased by 11.9 times.

**Conclusions:**

In our study, use of calcium channel blockers was associated with decreased in-hospital mortality in patients with COVID-19 infection. This finding was obtained by quickly adapting an NLP pipeline to the domain of the novel disease; the adapted pipeline still performed sufficiently to extract useful information. When that information was used to supplement existing structured data, the sample size could be increased sufficiently to see treatment effects that were not previously statistically detectable.

## Introduction

Outbreaks of novel diseases can create enormous strain on public health systems. Since the time of Snow's pioneering work [1] on the epidemiology of the London cholera outbreak of 1854, it has been clear that information is key to the successful abatement of these substantial public health challenges. Currently, health care systems have access to quantities of data that would have been unimaginable in Snow’s time. Because these data are in electronic format, they can be manipulated and exploited rapidly. However, a novel disease poses special challenges for informatics solutions. Biomedical informatics relies for the most part on structured data; structured data require a preexisting data or knowledge model; and a novel disease will not have a preexisting knowledge model. This poses a formidable obstacle to leveraging informatics solutions to address the type of public health crisis the world is facing at the time of writing. One solution to the lack of structured information is natural language processing (NLP).

Biomedical text mining, or the use of textual data, in electronic health records (EHRs) has often been proposed as a method for converting unstructured data to the structured data that is needed in public health informatics. One of the advantages of biomedical text mining is that it can be developed rapidly [2], which can permit the leveraging of electronic health records of patients with a novel disease as quickly as they are entered into the EHR. However, although this has often been suggested [3], there has never been an opportunity to actually test that claim in real time. Thus, the current novel coronavirus disease (COVID-19) pandemic, with all of its challenges, presents an opportunity to advance the state of public health informatics. In this paper, we tested this possibility with a case study on the effects of use of calcium channel blockers (CCBs) in patients with high blood pressure on the risk of death from COVID-19 infection. An association between CCB and the outcome of COVID-19 infection has already been suggested [4] but has not previously been explored in a large multicenter clinical study.

## Methods

### Data Source and NLP Pipeline

The data used in this study were obtained from 39 different hospitals in the Paris metropolitan area in the Assistance Publique – Hôpitaux de Paris (AP-HP) system. Focusing on this region of the country and on a large number of hospitals afforded a diversity of patient demographics that would not be available in most other parts of the country. As of May 4, 2020, the Entrepôt de Données de Santé (EDS)-COVID data set contained 84,966 electronic records of suspected or confirmed patients with COVID-19 (see Table 1 for further details on the data set). The records comprise structured fields and free text documents, including clinical notes and narratives. Most of the textual documents do not follow a specific structure and contain different types of patient information, such as patient history, family history, laboratory results, drug history, and prescriptions. Therefore, they represent an excellent test case for the real abilities of text mining. We used the following pipeline:

- Typical preprocessing steps (ie, text cleaning and sentence detection) were applied to the full data set (see Multimedia Appendix 1 for a detailed description).
- Drug names and details of administration (dose, route of administration, frequency, and duration) were extracted via a deep learning approach based on bidirectional encoder representations from transformers (BERT) contextual embeddings [5] (NLP Medication).
- Specific phenotypes associated with COVID-19 (eg, obesity, smoking status), scores (eg, sequential organ failure assessment score) and physiological measures (eg, BMI), were extracted via a list of 60 regular expressions (NLP RegExp).
- All signs, symptoms, and comorbidities included in the Unified Medical Language System (UMLS) [6] were extracted with the quickUMLS algorithm [7] (NLP UMLS).

A visual depiction of the pipeline is provided in Multimedia Appendix 2.

The NLP medication extraction model was a bidirectional long short-term memory with a conditional random field (BiLSTM-CRF) [8] layer on top of a vector representation of tokens using BERT [5]. We fine-tuned multilingual BERT on a set of 10 million clinical texts from EHRs. The model was trained on the APMed corpus, a manually annotated corpus of French clinical texts described in [9]. We used the FLAIR [10] implementation with 2 layers of 1024 units for the LSTMs with an asynchronous stochastic gradient descent (ASGD) optimizer and a reduction of the learning rate on plateau.

The NLP regular expression for the extraction of specific phenotypes was a set of 60 regular expressions developed manually and iteratively by medical informatics experts and physicians. We evaluated their precision at the sentence level using a random sample of 100 positive sentences for each regular expression. Examples of these expressions can be found in Multimedia Appendix 3.

All the terms extracted by the NLP pipeline, regardless of the method, were automatically annotated according to their modality (negated or hypothetical) and experiencer in the text, as described in previous work [11]. The outputs of the NLP pipeline were normalized to the Observational Medical Outcomes Partnership (OMOP) common data model (CDM) [12] and were fed back to the database system on a daily basis.

### Data Availability

Data supporting this study can be made available on request, on condition that the research project is accepted by the scientific and ethics committee of the AP-HP health data warehouse [13].

### Clinical Application: Long-Term CCB Use and Outcomes of COVID-19 in Patients With High Blood Pressure

The clinical goal of this case study was to evaluate the potential effects of CCBs on in-hospital mortality related to COVID-19 [4]. To achieve this goal, we used two different sources of data. The first source was two elements of structured data: International Classification of Disease, Tenth Revision (ICD-10) codes and medication prescriptions from an electronic prescription system. The second source was information on medications and comorbidities extracted by the NLP pipeline from nonstructured fields in the EHR. The inclusion criterion for patients was COVID-19 disease confirmed by reverse transcriptase–polymerase chain reaction (RT-PCR).

We considered a patient as receiving long-term treatment with CCBs (Multimedia Appendix 4) if there were at least two mentions (in structured data or extracted with NLP, respectively) in the last 6 months. We qualified cases as having comorbidities through one occurrence of an ICD-10 code (Multimedia Appendix 5) or two NLP mentions in the last 6 months.

The measured outcome was in-hospital mortality. We used a multivariate Cox proportional hazard model [14] that was adjusted according to age, gender, and the presence of obesity, diabetes, and cancer. The level of significance was set as *P=*.05, and all statistical tests were two-sided. We used R statistical software v.3.6.2 (R Project) with the Survival package.

## Results

### NLP Pipeline

As Table 1 shows, NLP markedly expanded the quantity of medication and phenotype information available for the analysis. The number of data points for medication increased by 7.2 times (*NLP medication*)⁄(*structured medication*), and the number of phenotypes increased by 15.2 times ((*NLP RegExp* + *NLP UMLS*)⁄(*ICD-10 codes*). Among the 84,966 patients with records present in the EDS-COVID cohort (Table 1), 45,593 (53.7%) contained drug information in their narrative EHR documents, whereas only 19,791 (23.3%) of the patients had medication information available in the structured fields in the EHR.

**Figure 1:**
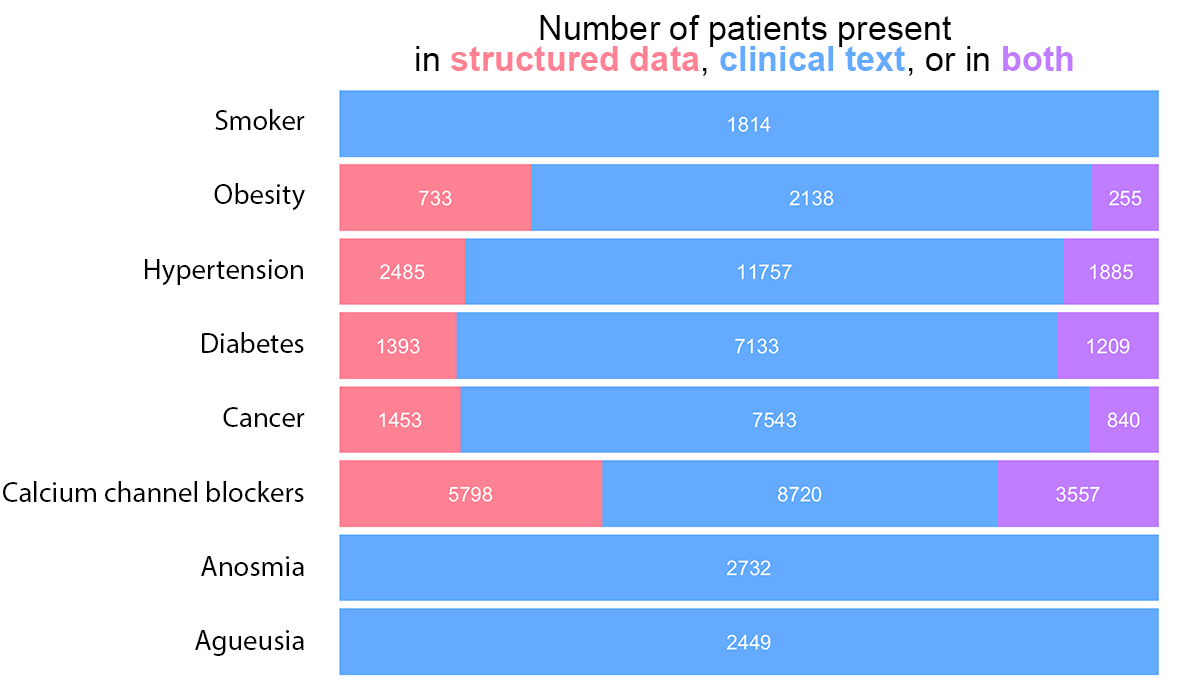
Quantity of patients with information for a selection of items depending on the source of data.

For specific phenotypes with existing ICD-10 codes (Figure 1), information was only available in clinical free-text fields for the majority of patients: 7133/8526 (60.2%) for diabetes, and 2138/2871 (74.5%) for obesity. Some items were absent from the structured data but could be recovered using the NLP extraction pipeline, such as COVID-19–specific symptoms such as ageusia (2449 patients) and anosmia (2732 patients).

In terms of quality, the extraction of medication names showed an F1 score of 93.8% (91.6% after normalization) in all sections. When focusing on the admission and discharge treatment sections, the F1 score was 96.7% (96.0% after normalization). The detailed results are shown in Multimedia Appendix 6. Regarding the phenotypes extracted by regular expressions in our case study, hypertension showed a precision of 99%, and obesity, diabetes, and cancer showed precisions of 94%, 80%, and 91%, respectively.

**Table 1:** Description of the information extracted using the NLP pipeline in the EDS-COVID cohort (N=84,966).

Source	Patient records (N=84,966), n (%)	Documents (N=1,524,057), n (%)	Data points, n
NLP^a^ Medication	45,593 (53.7)	696,125 (45.7)	5,995,945
NLP RegExp^b^	44,498 (52.4)	711,900 (46.7)	5,449,932
NLP UMLS^c^	44,035 (51.8)	833,610 (54.7)	19,626,172
Structured medication	19,791 (23.3)	N/A^d^	826,554
ICD-10^e^ codes	38,993 (45.9)	N/A	1,643,819

^a^NLP: natural language processing.

^b^RegExp: regular expression.

^c^UMLS: Unified Medical Language System.

^d^N/A: not applicable.

^e^ICD-10: International Classification of Disease, Tenth Revision.

### Case Study

**Figure 2:**
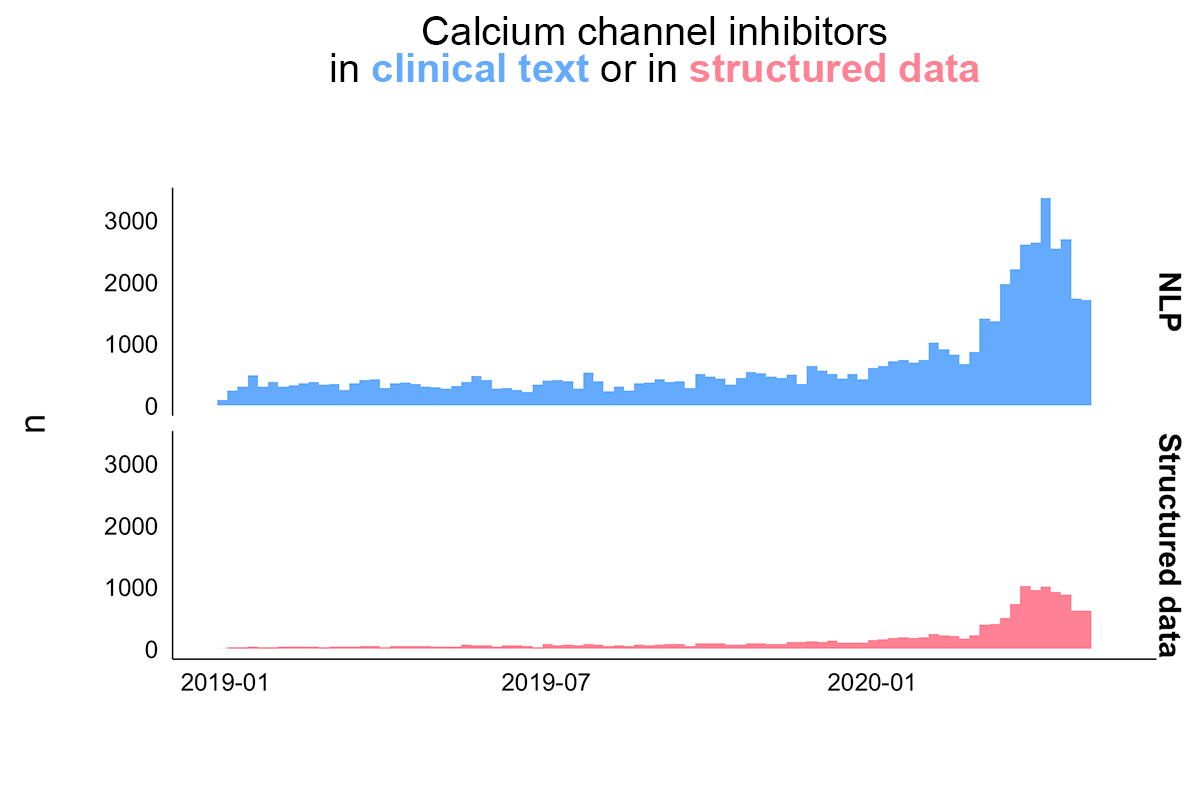
Quantity of information about calcium channel blockers for the two data sources over time. NLP: natural language processing.

Of the 84,966 total patients, 3965 (4.7%) were included using the NLP pipeline, of which only 1343 (15.9%) could be included if the study were limited to the use of structured data; this increased the number of patients added for the case study increased by 2.95 times (Multimedia Appendix 7). A detailed description of the population of patients who tested positive for COVID-19 with a history of high blood pressure can be found in Multimedia Appendix 8). In terms of the temporal depth of CCB treatment information, Figure 2 shows that a higher volume of information was obtained from text fields compared to structured data.

When using only structured data, we observed an adjusted hazard ratio (aHR) of 0.83 (95% CI 0.67-1.05) for treatment with CCBs; this result was not statistically significant (*P*=.12). When including NLP data, the aHR became 0.82 (95% CI 0.71-0.94), which represents a statistically significant reduction of the risk of death (*P*=.005). Similar results can be observed that support an increased risk of mortality with the presence of diabetes and cancer as comorbidities (Table 2).

**Table 2:** Results of the multivariate Cox survival model.

Characteristic		Structured data			NLP^a^		
		aHR^b^	95% CI	*P* value	HR^c^	95% CI	*P* value
Calcium channel blockers		0.83	0.67-1.05	.12	0.82	0.71-0.94	.005
**Age (years)**							
	45-64	Reference	N/A^d^	N/A	N/A	N/A	N/A
	18-44	0.20	0.03-1.46	.11	0.35	0.15-0.80	.01
	65-74	1.50	0.99-2.27	.053	1.95	1.54-2.47	<.001
	75-84	1.68	1.14-2.48	.009	2.94	2.35-3.69	<.001
	85+	2.45	1.66-3.61	<.001	3.99	3.16-5.03	<.001
**Gender**							
	Female	Reference	N/A	N/A	N/A	N/A	N/A
	Male	1.59	1.27-2.00	<.001	1.53	1.32-1.77	<.001
Obesity		1.07	0.81-1.42	.60	1.13	0.90-1.41	.30
Diabetes		1.22	0.98-1.52	.08	1.25	1.09-1.45	.002
Cancer		1.20	0.96-1.49	.11	1.34	1.15-1.56	<.001

^a^NLP: natural language processing.

^b^aHR: adjusted hazard ratio.

^c^HR: hazard ratio.

^d^N/A: not applicable.

## Discussion

In this paper, we investigated the potential utility of biomedical NLP in the context of a rapidly emerging novel disease. To do this, we asked a specific question: Does the leveraging of unstructured textual information via NLP yield clinically actionable information? To answer this question, we used NLP to extract information about hypertension and a medication for treating it from the EHRs of patients with COVID-19. The results showed that an NLP pipeline can be adapted quickly to the domain of a novel disease, it can perform well enough to extract useful information, and when that information is used to supplement the structured data that is already available, the sample size can be increased sufficiently to see treatment effects that were not previously statistically detectable.

Several agencies, notably the European Medicines Agency, have highlighted the benefits of using real-world data for research, in particular for the generation of complementary evidence and new hypotheses [15]. During the peak of the COVID-19 pandemic, the time available for clinicians to enter EHR data was greatly reduced. Medical informatics became vital to manage the crisis in hospitals and acquire better knowledge of the disease. The NLP pipeline was implemented within two weeks at the beginning of the COVID-19 epidemic in France, building on previous developments in artificial intelligence and text mining at AP-HP. More specifically, combining nonspecific preexisting developments (eg, negation, family history, and hypothesis detection) to tailored extractions (ie, regular expressions) allowed us to obtain rapid results of sufficient quality.

Approximately 60 internal research projects exploring EDS-COVID data were submitted for Institutional Review Board approval within the first eight weeks of COVID-19 epidemic. More than half of these projects studied variables such as symptoms (eg, ageusia), radiological signs (eg, crazy paving), comorbidities (eg, obesity), and drug history (eg, hydroxychloroquine), requiring extraction of information from narrative reports in EHRs.

The case study described in this paper shows the possible impact of using information extracted from text in the EHR for COVID-19 research. More precisely, the conclusions of the above study would have been different if information from unstructured fields had been excluded. In our case study, the addition of information from NLP did not dramatically change the hazard ratio from the analyses; however, it allowed us to include more patients and therefore narrowed the CIs and increased the statistical power. Note that the increased statistical power is mainly due to the increase in the number of patients included and the quantity of data available. Further analyses are required to assess the validity of the associations detected here, given that some confounding biases may remain and provoke false positive results. Reproducing the analysis with an external population or performing falsification testing [16] could help improve the validity of these findings.

## Appendix

Multimedia Appendix 1 Supplementary methods.

Multimedia Appendix 2 Description of the natural language processing pipeline.

Multimedia Appendix 3 Examples of regular expression for the extraction of phenotypes.

Multimedia Appendix 4 Definition of calcium channel blockers (name, ATC number).

Multimedia Appendix 5 Definition of phenotypes (name, ICD0-10 code).

Multimedia Appendix 6 Performance of the medication information extraction model before and after normalization of the entities.

Multimedia Appendix 7 Flowchart of the use case: patients who tested positive for COVID-19 who have hypertension.

Multimedia Appendix 8 Characteristics of the population of COVID positive patients with hypertension in EDS-COVID.

## Abbreviations

aHR: adjusted hazard ratio
AP-HP: Assistance Publique – Hôpitaux de Paris
ASGD: asynchronous stochastic gradient descent
BiLSTM-CRF: bidirectional long short-term memory with a conditional random field
CCB: calcium channel blocker
CDM: common data model
COVID-19: coronavirus disease
EDS: Entrepôt de Données de Santé
EHR: electronic health record
ICD-10: International Classification of Disease, Tenth Revision
NLP: natural language processing
RT-PCR: reverse transcriptase–polymerase chain reaction
OMOP: Observational Medical Outcomes Partnership
UMLS: Unified Medical Language System
