# Supplementary material for: Natural Language Processing for Rapid Response to Emergent Diseases: Case Study of Calcium Channel Blockers and Hypertension in the COVID-19 Pandemic
Source: J Med Internet Res. 2020 Aug 14;22(8):e20773. doi: 10.2196/20773 (PMC7431235; doi:10.2196/20773)
Supplement: Multimedia Appendix 1 [file jmir_v22i8e20773_app1.docx]

**The 8 steps from the narrative reports to the OMOP Note NLP table**

As described in Multimedia Appendix 2, we applied pre-processing treatments to clean the narrative reports before the extraction of phenotypes and scores.

1- In order to properly detect the structure of the reports, we have to manage false end of line added by the conversion of the narrative records from pdf or simple text file.

2- The pipeline first replaces accurately end lines in the narrative reports by a full stop or a space to reconstruct the sentences.

3- Then it classifies the sentences according to the certainty (i.e. hypothesis or certain), and the experiencer (i.e. family history or patient).  For example, the sentence “In case of fever, take 1g of acetaminophen” is classified as “hypothesis” and “patient”.

4- The algorithm cuts the sentences into syntagms and classifies them according to their polarity (“negation” or “affirmation”). The syntagms are stored along with their position in the text.

5- We applied the extraction of phenotypes on these syntagms by using the regular expression defined by experts. A set of 60 comorbidities and values defined by experts (json definition of rules, format, version, exclusion criteria). See example in Multimedia Appendix 3.

6- On the spans from step 4, we also applied the QuickUMLS algorithm. This algorithm detects in the text, concepts from the UMLS using approximate string matching. We limited the concepts to the semantic group Disorders of the UMLS 2019AA.

We obtained for each method a list of phenotypes and scores and the modified inherited from the syntagm (certainty, experiencer, polarity).

7- The extraction of a drug mention and its attributes of administration (i.e., dose, frequency, duration, condition of intake) were performed using a couple of deep learning models based on BERT contextual embeddings^1^ : one for the entities and one for the relations between entities. The model was trained on the APmed corpus^2^, a previously annotated dataset for this task, was evaluated at 94% F-mesure for drug, 69% F-mesure for drug class, 93% F-mesure for dose, and 93% F-mesure for frequencies. Extracted drug mentions were then normalized to the ATC terminology using approximate string matching.

8- The final step consists in formatting and standardizing all the extracted information into the note_nlp table of the OMOP common data model.
