# Supplementary figures and images for: Natural Language Processing for Rapid Response to Emergent Diseases: Case Study of Calcium Channel Blockers and Hypertension in the COVID-19 Pandemic
Source: J Med Internet Res. 2020 Aug 14;22(8):e20773. doi: 10.2196/20773 (PMC7431235; doi:10.2196/20773)

**eFigure 1: Description of the natural language processing pipeline**


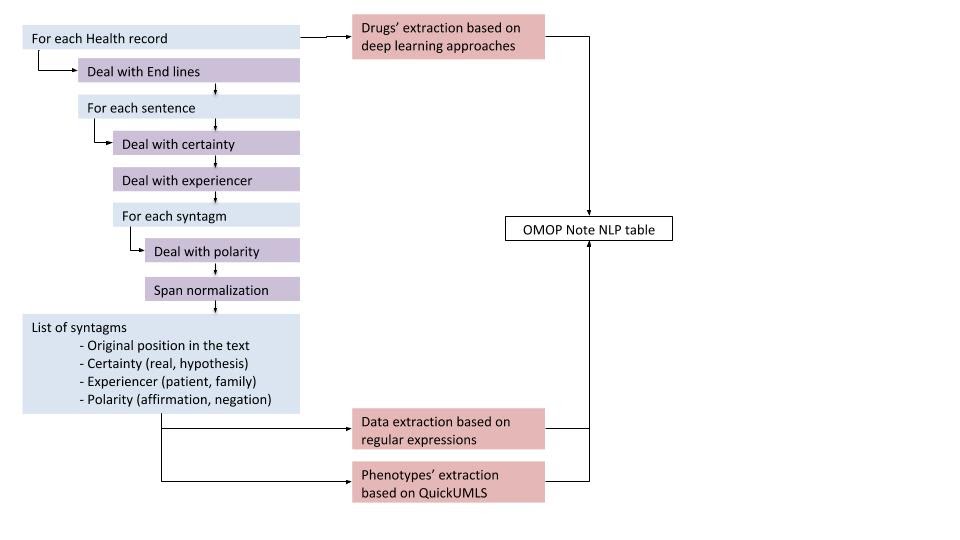

Supplement: Multimedia Appendix 2 [file jmir_v22i8e20773_app2.docx]

**eFigure 3: Flowchart of the use case: COVID positive patients with hypertension**


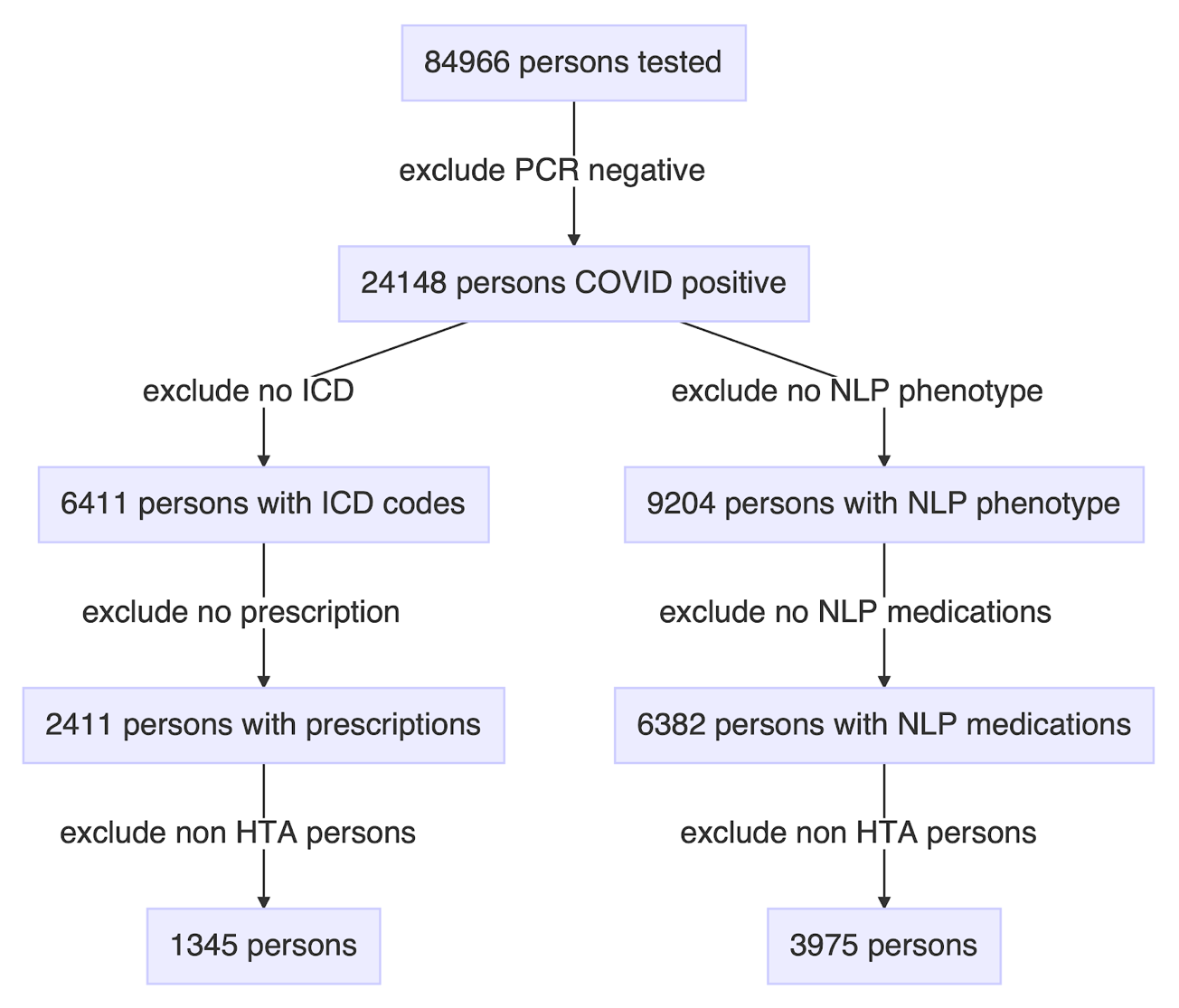

Supplement: Multimedia Appendix 7 [file jmir_v22i8e20773_app7.docx]
