# Supplementary material for: Natural Language Processing for Rapid Response to Emergent Diseases: Case Study of Calcium Channel Blockers and Hypertension in the COVID-19 Pandemic
Source: J Med Internet Res. 2020 Aug 14;22(8):e20773. doi: 10.2196/20773 (PMC7431235; doi:10.2196/20773)
Supplement: Multimedia Appendix 3 [file jmir_v22i8e20773_app3.docx]

**eFigure 2: Examples of regular expression for the extraction of phenotypes**

{"libelle":"BPCO, maladie pulmonaire, pneumopathie, HTAP",

    "regexp":"[^a-z]BPCO[^a-z]|[^a-z]HTAP[^a-z]|bronchopneumopathie|maladie.{0,5}pulmonaire|pneumopathie|a[sth][sth][sth]me|bronch?ospasme|a[sth][sth][sth]matique|insuf.{1,2}isance.{0,5}respiratoire.{0,5}chronique|emph.s[\u00e8e]me|emf.s[\u00e8e]me",

    "id_regexp":"id_regexp_bpco",    "list_cui":"C0024117,C0024115,C0032285,C0020542,C0004096,C0006266,C0264492,C0034067",

    "regexp_exclude":"",

    "version":"v2",

    "filter_document":"",

    "date_modification":"12\/05\/2020",

    "deprecated":"false",

    "comment":"",

    "refresh":"false"}

{"libelle":"Cancer",

    "regexp":"(?<!pre)(?<!pre )cancer[^a-z]|tumeur(?!.{0,7}benigne)|carcinome|m[e\u00e9]lanome|n[e\u00e9]oplasie|sarcome",

    "id_regexp":"id_regexp_cancer",

    "list_cui":"C0027651,C1882062,C0006826",

    "regexp_exclude":"",

    "version":"v2",

    "filter_document":"",

    "date_modification":"12\/05\/2020",

    "deprecated":"false",

    "comment":"",

    "refresh":"false"},
