# Supplementary material for: Natural Language Processing for Rapid Response to Emergent Diseases: Case Study of Calcium Channel Blockers and Hypertension in the COVID-19 Pandemic
Source: J Med Internet Res. 2020 Aug 14;22(8):e20773. doi: 10.2196/20773 (PMC7431235; doi:10.2196/20773)
Supplement: Multimedia Appendix 4 [file jmir_v22i8e20773_app4.docx]

**eTable 1: Definition of calcium channel blockers (name, ATC)**

| Amlodipine | C08CA01 |
| --- | --- |
| Diltiazem | C05AE03\|C08DB01 |
| Felodipine | C08CA02 |
| Isradipine | C08CA03 |
| Lacidipine | C08CA09 |
| Lercanidipine | C08CA13 |
| Manidipine | C08CA11 |
| Nicardipine | C08CA04 |
| Nifedipine | C08CA05 |
| Nitrendipine | C08CA08 |
| Verapamil | C08DA01 |
