# Supplementary material for: Natural Language Processing for Rapid Response to Emergent Diseases: Case Study of Calcium Channel Blockers and Hypertension in the COVID-19 Pandemic
Source: J Med Internet Res. 2020 Aug 14;22(8):e20773. doi: 10.2196/20773 (PMC7431235; doi:10.2196/20773)
Supplement: Multimedia Appendix 5 [file jmir_v22i8e20773_app5.docx]

**eTable 2: Definition of phenotypes (name, ICD10 codes)**

| Cancer | all codes from Chapter II |
| --- | --- |
| diabetes | E10, E11, E12, E13, E14 |
| High blood pressure | I10, I15 |
| obesity | E66 |
