# Supplementary material for: Natural Language Processing for Rapid Response to Emergent Diseases: Case Study of Calcium Channel Blockers and Hypertension in the COVID-19 Pandemic
Source: J Med Internet Res. 2020 Aug 14;22(8):e20773. doi: 10.2196/20773 (PMC7431235; doi:10.2196/20773)
Supplement: Multimedia Appendix 6 [file jmir_v22i8e20773_app6.docx]

**eTable 3A: Performance of the medication information extraction model BEFORE normalization of the entities.**

| **Entities** | **Precision**  (% [90% CI]) | **Recall**  (% [90% CI]) | **F-measure**  (% [90% CI]) |
| --- | --- | --- | --- |
|  |  |  |  |
| **All sections** | | | |
| Drug name | 92.1 [89.5-94.5] | 95.6 [93.3-97.4] | 93.8 [92.1-95.5] |
| Dose | 93.0 [90.0-95.9] | 93.9 [91.0-96.5] | 93.4 [91.3-95.5] |
| Frequency | 94.2 [91.4-96.9] | 92.6 [88.9-95.6] | 93.4 [91.0-95.5] |
| **Sections admission or discharge treatment** | | | |
| Drug name | 94.6 [90.7-98.0] | 98.9 [96.8-100.0] | 96.7 [94.3-98.7] |
| Dose | 97.6 [94.4-100.0] | 97.4 [94.2-100.0] | 97.5 [95.2-99.4] |
| Frequency | 97.3 [93.9-100.0] | 97.3 [93.7-100.0] | 97.3 [94.9-99.3] |

**eTable 3B: Performances of the medication information extraction model AFTER normalization of the entities.**

| **Entities** | **Precision**  (% [90% CI]) | **Recall**  (% [90% CI]) | **F-measure**  (% [90% CI]) |
| --- | --- | --- | --- |
|  |  |  |  |
| **All sections** | | | |
| Drug name | 99.2 [98.0-100.0] | 85.1 [81.4-88.7] | 91.6 [89.3-93.7] |
| Dose | 92.9 [89.9-95.8] | 92.4 [89.4-95.2] | 92.7 [90.4-94.7] |
| Frequency | 95.1 [92.1-97.6] | 89.6 [85.6-93.2] | 92.2 [89.7-94.5] |
| **Sections admission or discharge treatment** | | | |
| Drug name | 100 | 92.4 [87.3-97.1] | 96.0 [93.2-98.5] |
| Dose | 97.4 [94.2-100.0] | 97.5 [94.3-100.0] | 97.5 [95.1-99.4] |
| Frequency | 97.2 [93.5-100.0] | 93.3 [88.0-98.4] | 95.1 [91.7-98.1] |
