# Supplementary material for: Natural Language Processing for Rapid Response to Emergent Diseases: Case Study of Calcium Channel Blockers and Hypertension in the COVID-19 Pandemic
Source: J Med Internet Res. 2020 Aug 14;22(8):e20773. doi: 10.2196/20773 (PMC7431235; doi:10.2196/20773)
Supplement: Multimedia Appendix 8 [file jmir_v22i8e20773_app8.docx]

**eTable 4: Characteristics of the population of COVID positive patients with hypertension in EDS-COVID**. NLP: Natural language Processing

| Characteristic | **NLP**, N = 3965^1^ | **Structured,** N = 1343^1^ |
| --- | --- | --- |
| **Age** |  |  |
| 45-64 | 1070 (27%) | 205 (15%) |
| 18-44 | 175 (4.4%) | 29 (2.2%) |
| 65-74 | 913 (23%) | 252 (19%) |
| 75-84 | 925 (23%) | 392 (29%) |
| 85+ | 882 (22%) | 465 (35%) |
| **Death** | 810 (20%) | 340 (25%) |
| **Gender** |  |  |
| Female | 1729 (44%) | 677 (50%) |
| Male | 2236 (56%) | 666 (50%) |
| **Cancer** | 886 (22%) | 444 (33%) |
| **Diabetes** | 1676 (42%) | 560 (42%) |
| **Obesity** | 518 (13%) | 286 (21%) |
| **Calcium channel blockers** | 1846 (47%) | 525 (39%) |
| ^1^Statistics presented: n (%) | | |
